# Sysmex XN‐Based Evaluation of the Diagnostic Performance of High‐Fluorescent Cells From CSF as a Supportive Diagnostic Criterion in Neurological Diseases

**DOI:** 10.1111/ijlh.14466

**Published:** 2025-03-20

**Authors:** Benedict Schwarz, Christopher Hardt, Katharina Friedrich, Monika Prpic, Anja Osterloh, Frank L. Heppner, Klemens Ruprecht, Kai Kappert, Amir Jahic

**Affiliations:** ^1^ Institute of Diagnostic Laboratory Medicine, Clinical Chemistry and Pathobiochemistry, Charité—Universitätsmedizin Berlin Corporate Member of Freie Universität and Humboldt—Universität Zu Berlin Berlin Germany; ^2^ Laboratoriumsmedizin & Toxikologie Labor Berlin—Charité Vivantes GmbH Berlin Germany; ^3^ Institute of Pathology Ulm University Ulm Germany; ^4^ Department of Neuropathology Charité—Universitätsmedizin Berlin, Corporate Member of Freie Universität and Humboldt—Universität Zu Berlin Berlin Germany; ^5^ Department of Neurology Charité—Universitätsmedizin Berlin, Corporate Member of Freie Universität and Humboldt—Universität Zu Berlin Berlin Germany

**Keywords:** cerebrospinal fluid, clinical patient stratification, high‐fluorescent cells, mathematical disease modelling, Sysmex XN hematology analyzer

## Abstract

**Introduction:**

Automatic cytological analysis of cerebrospinal fluid (CSF) by Sysmex XN‐Series represents a convenient laboratory platform for quantitative examination of nucleated CSF cells (monomorphonuclear (MN), polymorphonuclear (PMN), high‐fluorescent (HF)). HF cells (HFC), a research laboratory parameter so far, seem to be associated with certain clinical patterns. Hence, we aimed to determine the diagnostic HFC value for different clinical categories in neurological settings.

**Methods:**

Morphological classification of automatically detected HFC was carried out using manual light microscopy. Automatic method precision for cell differentiation was evaluated in comparison. In 284 cases, multiple correlation strategies and mathematical disease modellings enabled an explorative analysis of HFC suitability for case stratification into the categories Hemorrhage, Inflammation, Neoplasia, other, and unknown.

**Results:**

Manual microscopic reevaluation revealed plasma cells, macrophages, and malignant cells being HFC correlates in 80% of automatically detected HFC. Method correlation for automatic and manual CSF cell differentiation approaches was 95%, yielding a negative bias of 4.3% for MN and positive bias of 0.4% and 3.9% for PMN and HF, respectively. When HFC were used as a “stand‐alone” predicting tool, diagnostic accuracy, specificity, and sensitivity depended on the clinical condition, ranging from > 0.5 up to > 0.7 for Hemorrhage, Inflammation, and Neoplasia. However, multiparametric correlation analyses combining laboratory CSF diagnostics and mathematical methods defined HFC as a relevant laboratory parameter for adequate clinical case stratification. With an expected random distribution of 25% for four clinical categories, almost 70% of cases were correctly classified when the HFC‐based mathematical algorithm was applied.

**Conclusion:**

HFC has significant diagnostic and/or predictive value for neurological diseases.

## 1 Introduction

Laboratory examination of cerebrospinal fluid (CSF) plays a crucial role in the diagnosis and management of various neurological diseases [1]. Standard laboratory CSF parameters, glucose, lactate, and total protein (TP) are regularly quantified and clinically interpreted in cases of central nervous system (CNS) hemorrhage, inflammation, malignancy, and others [2, 3]. Further laboratory CSF parameters such as oligoclonal bands (OCB) and/or the presence of intrathecal immunoglobulins (Ig) indicate blood brain barrier (BBB) disturbances and are assessed in the light of specific diagnostic questions [4, 5, 6, 7, 8, 9]. However, total cell count (TCC) as well as the corresponding cytological composition of CSF remains essential for clinical diagnostic decisions [10]. Although an increased TCC or the presence of certain nucleated cell types in CSF cannot always provide a final diagnosis, they can assist as an important supportive diagnostic clue for conditions such as CNS associated hemorrhage [11], inflammation [1, 11, 12], or malignancy [13].

The gold standard laboratory approach for cytological CSF differentiation still remains the manual light microscopy technique, even though this method requires trained personnel and is characterized by a high temporal and financial expenditure [14]. Modern automatic hematology analyzers like Sysmex XN‐Series, on the other hand, also perform fast and accurate TCC, making at the same time diagnostic suggestions for specific cell types present in CSF [15]. By using an automatic fluorescence‐based approach, nucleated cells from CSF are classified by a hematology analyzer as monomorphonuclear (MN: lymphocytes, monocytes), polymorphonuclear (PMN: granulocytes) and high‐fluorescent (HF: e.g., plasma cells, macrophages including erythro‐/hemosiderophages, and malignant cells) [15].

Several studies have already evaluated the diagnostic performance of automatic CSF cell differentiation in comparison to the manual light microscopy approach and have shown acceptable diagnostic correlations for CSF cell groups of MN and PMN [14, 15, 16, 17, 18, 19, 20, 21, 22, 23]. The CSF cell type of automatically detected HF cells (HFC), that is usually present in hemorrhage [11], inflammation [1, 12], and/or malignancy [13], is currently only considered as a research laboratory parameter and is usually not reported to clinicians. As CSF HFC predominantly represents plasma cells [14, 24, 25], macrophages [14, 19, 26], or malignant cells [27], they may have diagnostic relevance and therefore could serve as a further supportive laboratory parameter in certain clinical settings. However, the diagnostic link between automatically identified HFC in CSF and the underlying neurological diseases has only been studied little.

Thus, this study explored the diagnostic relevance of CSF HFC at the clinical laboratory interface for defined neurological conditions, considering automatic and manual (microscopic) cytological CSF examination. Moreover, based on a multiparametric approach and applying machine‐learning methods, we aimed at improving diagnostic capability by quantifying HFC from CSF in specific clinical settings.

## 2 Materials and Methods

### 2.1 CSF Samples and Data Set Collection

The study was approved by the ethics committee of the Charité—Universitätsmedizin Berlin (EA1/059/22) and the study was conducted in accordance with the Declaration of Helsinki (as revised in 2013). Informed consent was obtained from all individuals included in this study or their legal guardians or wards. Between June and September 2021, HFC were automatically detected in CSF samples from 149 cases of adult patients at Charité—Universitätsmedizin Berlin. All 149 HFC positive (HFC ≥ 1 cell/μL) cases were included in the study. From the same period, 135 CSF samples without evidence of HFC (HFC negative cases; HFC < 1 cell/μL) that were analyzed by an automatic hematology system were randomly selected and defined as a control group. Besides HFC status, the only inclusion criteria were the collection of CSF and corresponding serum samples. Laboratory data from serum was used for Reiber scheme calculations. CSF and blood (i.e., serum) sampling was performed under routine clinical conditions. Vacuum serum tubes (VACUETTE, Greiner Bio‐One GmbH, 72636 Frickenhausen, Germany) containing clot activator were filled and processed according to the manufacturer's recommendation. CSF and serum samples were stored at room temperature prior to analysis. Laboratory analyses were performed at the Labor Berlin—Charité Vivantes GmbH under routine conditions immediately after CSF and serum sample collections.

Due to missing data for certain laboratory CSF parameters, numbers decreased from an initial 150 cases per group. For both groups, HFC positive (HFC+) and HFC negative (HFC–), only the respective first CSF sample taken after admission to the hospital was considered. Laboratory data were collected from the laboratory information system (David, Medat Computersysteme GmbH, 80 636 München, Germany). Clinical data were collected from the clinical information system (SAP Medical Record, SAP SE, 69190 Walldorf, Germany). Based on information obtained from discharge letters of both groups, HFC+ and HFC–, five clinical categories Hemorrhage, Inflammation, Neoplasia, other and unknown were defined, and comparative analyses of laboratory data were carried out.

### 2.2 Numerical CSF Examination

CSF parameters glucose, lactate, and total protein were measured using the clinical chemistry analyzer Cobas8000 (Roche Diagnostics GmbH, 68 305 Mannheim, Germany). A nephelometric BN II laboratory system (Siemens Healthcare Diagnostics GmbH, 65 760 Eschborn, Germany) was used for analyses of albumin and immunoglobulins (IgA, IgG, IgM) from both CSF and serum. For detection of oligoclonal bands (OCB) a hydragel CSF isofocusing assay (Sebia Holding GmbH, 36 041 Fulda, Germany) was applied.

### 2.3 Cytological CSF Examination

Automatic CSF sample analyses for TCC and subsequent differentiation into MN, PMN, and HF cell types were carried out by a Sysmex XN‐Series hematology analyzer (Sysmex Deutschland GmbH, 22 848 Norderstedt, Germany) in body fluid mode.

Manual CSF cell differentiation was performed using May‐Gruenwald‐Giemsa stained cytospin preparations according to Zimmermann et al. [27]. To examine the CSF specimens, the Axiolab 5 microscope (Carl Zeiss Microscopy Deutschland GmbH, 73 447 Oberkochen, Germany) at 40× magnification was used at the Department of Neuropathology, Charité—Universitätsmedizin Berlin. Depending on cell density, 100–500 nucleated cells per cytospin were scanned and differentiated into lymphocytes (including activated lymphocytes), monocytes (including activated monocytes), granulocytes, plasma cells, macrophages (including erythro−/hemosiderophages) as well as malignant/tumor cells.

For comparative analysis of automatic and manual CSF cell differentiation, the cells selected by light microscopy as lymphocytes, including activated lymphocytes, and monocytes, including activated monocytes, were summarized as the MN cell population. The granulocytes were defined as the PMN cell population. Plasma cells, macrophages, including erythro−/hemosiderophages, as well as malignant/tumor cells, were subsumed as the HFC population.

### 2.4 Statistics

Statistical analysis was carried out using the Python libraries statsmodels and scikit‐learn. For the identification of systemic biases, we applied the Bland–Altman analysis. This approach calculated the difference between the manual and the automatic machine count against the average of the two methods for each cell subtype (MN, PMN, HF), with ± 1.96 times the standard deviation and representing the 95% confidence interval. Associations between ordinal variables were examined using the Mann–Whitney *U* test, and associations between categorical variables were examined using chi‐squared and Barnard's Exact tests.

Data were selected according to the presence or absence of HFC population (HFC+ vs. HFC–) followed by further classification into Hemorrhage, Inflammation, Neoplasia, other, and unknown clinical groups. Statistical differences between HFC+ and HFC– cases were analyzed across all five clinical categories; variables with more than 70% incomplete data were discarded. For variables with non‐normal distribution (Shapiro *p* > 0.05), the Mann–Whitney *U* test (numerical variables) and the chi‐squared test (categorical variables) were performed. To compare clinical categories for HFC+ cases, ANOVA tests were performed. Variables with significant results (*p* ≤ 0.05) underwent a Tukey's post hoc test enabling a direct comparison between clinical categories.

### 2.5 Integrative Analyses by Machine Learning Models

Variables with complete data set, numerical as well as categorical, were standardized and used as assessment parameters for a 10‐fold cross‐validated linear support vector machine classifier to determine feature importance (scikit‐learn's LinearSVC with the following hyperparameters: random_state = 0, tol = 1e‐4, multi_class = “ovr,” max_iter = 10 000, class_weight = “balanced”). The defined disease categories were used as categorical targets for the classifier.

The same parameter set was used for the configuration of a decision tree classifier, albeit leaving out the clinical category unknown; specific decision tree relevant hyperparameters (tree depth *d* = 3, 10‐fold cross validation) were defined beforehand.

The class imbalance among all four diagnosis groups (Hemorrhage, Inflammation, Neoplasia, other) was considered when composing training and test sets, as well as when scoring the balanced accuracy according to the literature [28].

## 3 Results

### 3.1 Characteristics of Studied Population

Based on clinical case evaluation at hospital admission, as well as diagnosis on discharge, all 284 cases (149 HFC+, 135 HFC–) were grouped into five clinical categories: Hemorrhage, Inflammation, Neoplasia, others, and unknown (Tables S1 and S2). Analysis of population characteristics such as age and sex revealed no significant differences comparing HFC+ and HFC– cases (Figure S1A,C). Also, the distribution of age and sex across the five categories showed no statistical significance between HFC+ and HFC– groups (Figure S1B,D). These data suggest a representative and well‐balanced study population for further analysis.

### 3.2 Definition of HFC Morphology and Their Distribution Across Clinical Categories

**FIGURE 1:**
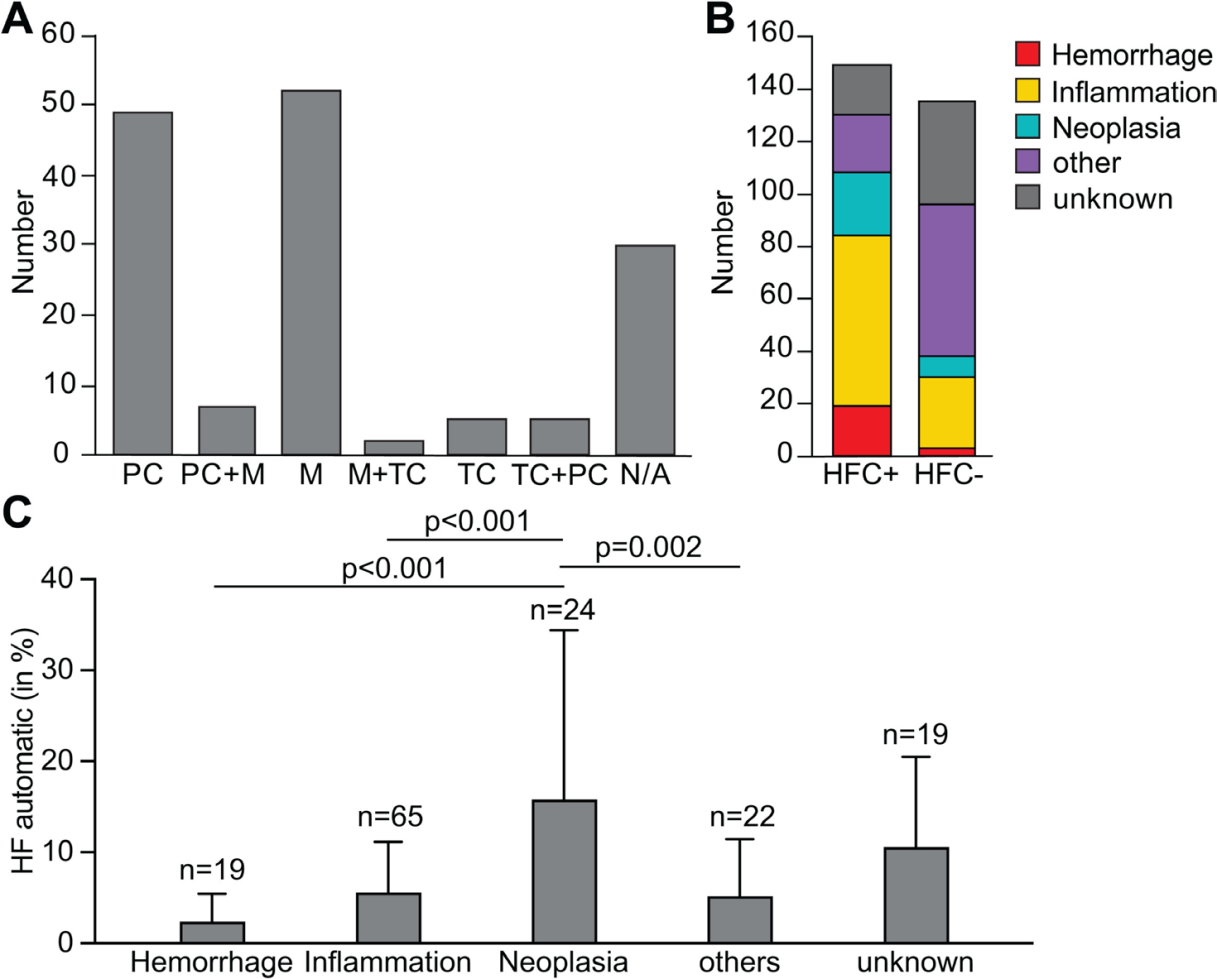
Fraction and morphology of HFC and their distribution across clinical categories. (A) Number of HFC+ cases classified into different morphology groups. M, macrophages; N/A, none of these; PC, plasma cells; TC, tumor cells. (B) Distribution of clinical categories in HFC+ and HFC– cases. HFC, high‐fluorescent cells. (C) Percentage of automatically counted HFC in HFC+ cases grouped into clinical categories. *p*, *p*‐value of Tukey's statistical significance test. See ANOVA results in Table S7.

To define the morphology of automatically detected HFC by the Sysmex XN‐Series hematology analyzer, we applied manual microscopic cytology of corresponding CSF cytospin preparations. We found plasma cells, macrophages, tumor cells, or a combination of those in ~80% (119/149) of HFC+ cases (Figure 1A, Table S3). In 30/149 automatically identified HFC+ cases (~20%) the light microscopy could not detect any HFC correlates. Considering all five clinical categories, most HFC+ cases (~72%) belong to the category of Inflammation (65/149; ~44%), followed by Hemorrhage (19/149; ~13%) and Neoplasia (24/149; ~16%) (Figure 1B, Table S4). However, ~15% (22/149) and ~13% (19/149) of HFC+ cases fell in the categories of other and unknown, respectively. A subsequent within‐category statistical analysis showed significant differences when comparing HFC+ and HFC– cases (Table S4). Strikingly, within the HFC+ cohort, HFC amount was significantly highest in the group of Neoplasia (Figure 1C).

### 3.3 Comparison of Automatic and Manual CSF Cell Differentiation Approaches

**FIGURE 2:**
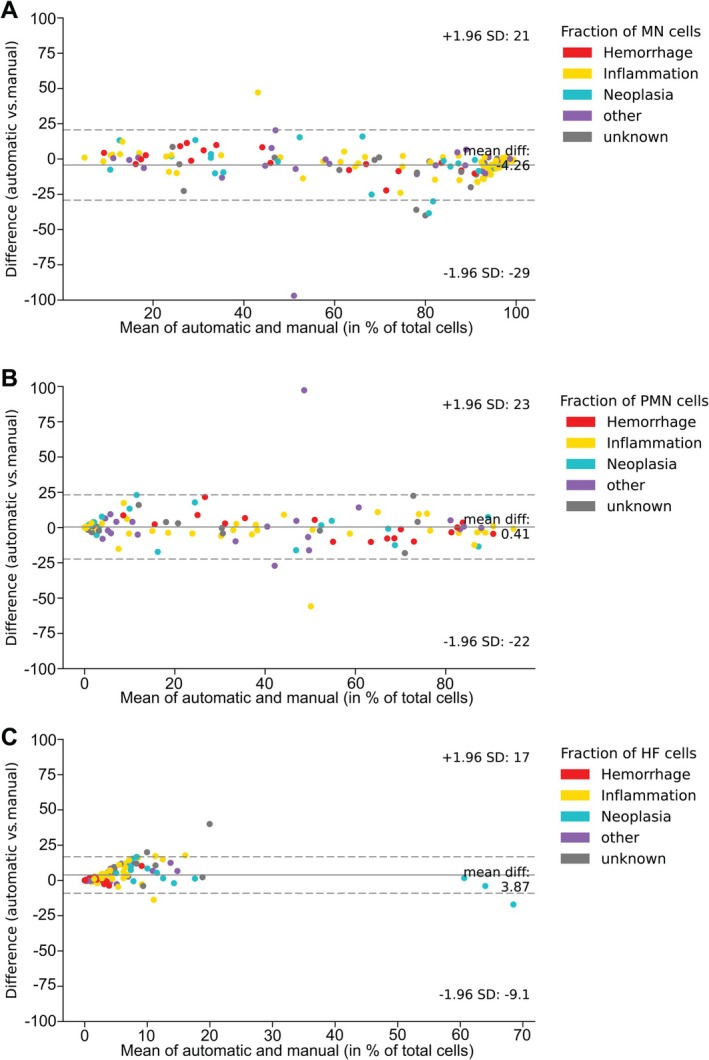
Evaluation of cytological performance of automated HFC quantification. Bland–Altman‐Plots showing the difference between automatic (hematology analyzer) and manual light microscopic quantification for monomorphonuclear (MN) cells (A), polymorphonuclear (PMN) cells (B) and high‐fluorescent (HF) cells (C). Continuous horizontal line, mean difference; dotted horizontal line, mean difference ± 1.96 times the standard deviation (SD).

To the best of our knowledge, a diagnosis‐based evaluation of the cytological CSF associated performance for a hematology analyzer has not yet been conducted. Therefore, we compared the results from automatic and manual CSF cell differentiation for all clinical categories. For all three CSF cell types characterized (MN, PMN, and HF), the comparative analysis showed an acceptable correlation with ~95% of the values being within the limits of agreement of two standard deviations (Figure 2). While for MN detection, the automatic method gave a slightly lower count (automatic < manual: 4.26%, Figure 2A), the automatic and manual approach for PMN quantification was almost identical (automatic > manual: 0.41%, Figure 2B), and for HFC detection, a slightly higher count was measured with the automatic method (automatic > manual: 3.87%, Figure 2C).

### 3.4 Determining Clinical Diagnosis Based on HFC Positivity

We used confusion matrices to evaluate the usability of the automated detection of HFC positivity for case stratification into the clinical categories Hemorrhage, Inflammation, and Neoplasia. Comparing the number of actual cases (based on diagnoses obtained from discharge letters) with the number of automatically classified cases resulted in a moderately high sensitivity from ~71% up to ~86%, indicating a high number of true positives. On the other hand, we observed only a moderate accuracy from ~53% up to ~61% that suggests a limited number of correct clinical classifications into the respective disease group when the total number of cases was considered (Table S5). Yet, for each clinical group, the ratio of true positive cases to the cases classified as positive for a diagnosis resulted in a low positive predictive value (PPV: < 0.4). The negative predictive value (NPP: > 0.8) was high for all clinical categories tested, showing that the case cohort without Hemorrhage, Inflammation, and Neoplasia is highly overlapping with the HFC– case cohort.

### 3.5 Multiparametric Correlation Analyses of HFC+ and HFC− Cases

**FIGURE 3:**
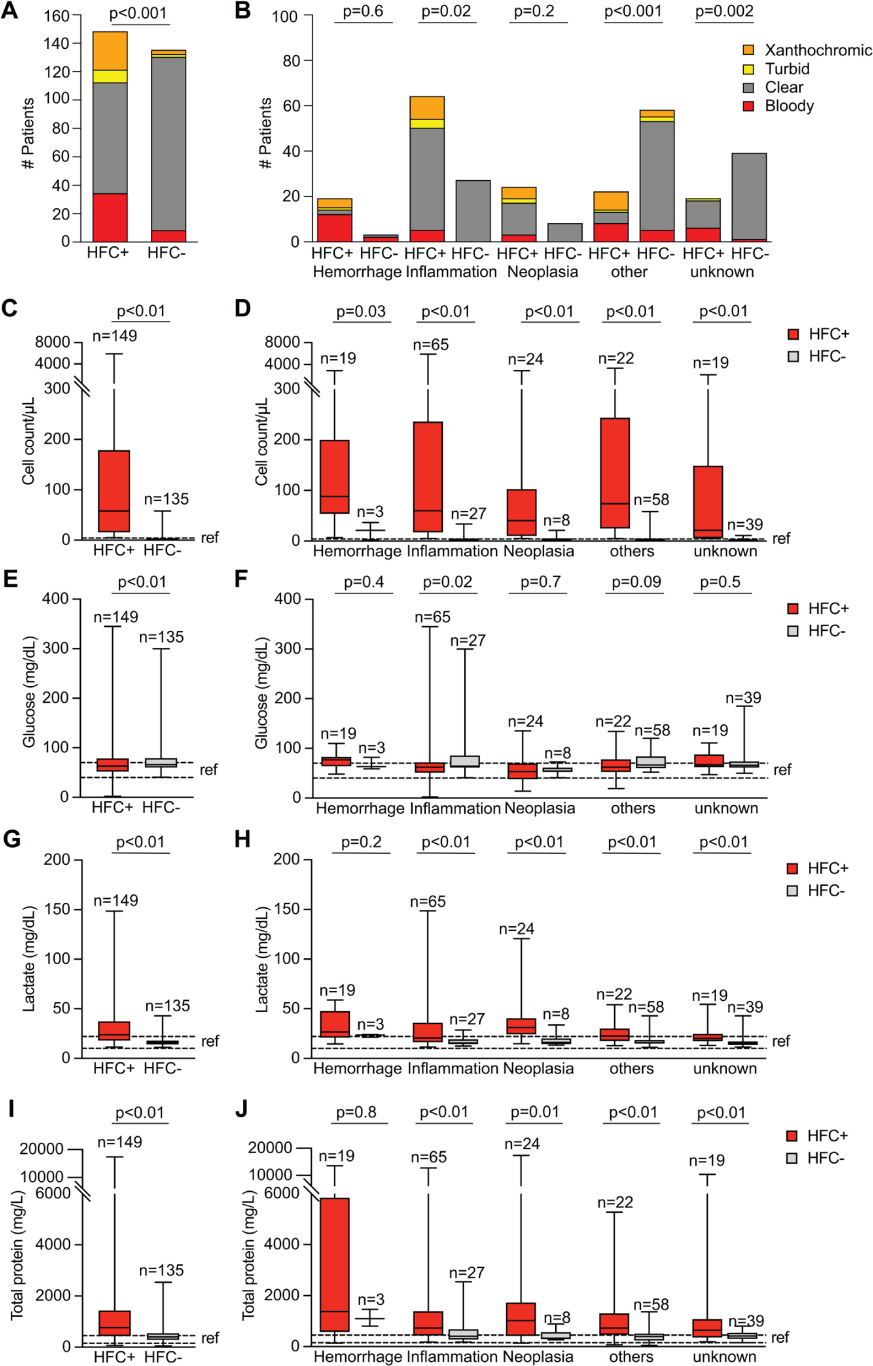
Diagnosis‐based multiparametric analysis comparing HFC+ and HFC– cases. (A, B) Differences in the macroscopic appearance of CSF between HFC+ and HFC– cases (A) as well as comparison within individual clinical categories (B). *p* represents statistical significance testing using chi squared test. (C, D) Analysis of differences in total cell count of CSF probes in HFC+ compared to HFC– cases (C) as well as comparison of HFC+ with HFC– cases grouped into clinical categories (D). (E–J) Analysis of differences in CSF concentrations of glucose (E, F), lactate (G, H), and total protein (I, J), comparing HFC+ with HFC– cases (E, G, I) as well as comparison within individual clinical categories (F, H, J). *n* represent number of cases per group; *p*, *p*‐value of statistical significance testing using Mann–Whitney *U* test. ref., reference range. See ANOVA results in Table S7.

Applying several diagnosis‐based multiparametric analyses, we tested the diagnostic applicability of HFC. We focused on routine CSF laboratory parameters such as macroscopic appearance, TCC, glucose, lactate, and TP, comparing HFC+ and HFC– cases and considering all five clinical categories. Compared to the HFC– group, with most cases having clear, colorless CSF specimen, there was a significant increase in the number of cases with bloody, turbid, or xanthochromic CSF specimen in the group of HFC+ cases, also when each clinical category was separately considered (Figure 3A,B, Table S6 for % of difference and statistical significance). The comparative analysis of clinical chemistry CSF parameters between HFC+ and HFC− groups showed a significant increase of TCC in total and for all five categories in HFC+ (Figure 3C,D, Table S6). There was a significant decrease of glucose levels in total and for Inflammation in HFC+ (Figure 3E,F, Table S6) and a significant increase of lactate in total and for Inflammation, Neoplasia, others, and unknown in HFC+ (Figure 3G,H, Table S6). Furthermore, there was a significant increase of TP in total and for Inflammation, Neoplasia, others, and unknown in HFC+ (Figure 3I,J, Table S6).

Additional clinical analyses in the context of symptom presentation revealed that the detection of HFC positively correlated with headache symptoms but neither with the presentation of motor nor sensory symptoms (Figure S2).

### 3.6 Reiber Schemes Associated HFC Evaluation

**FIGURE 4:**
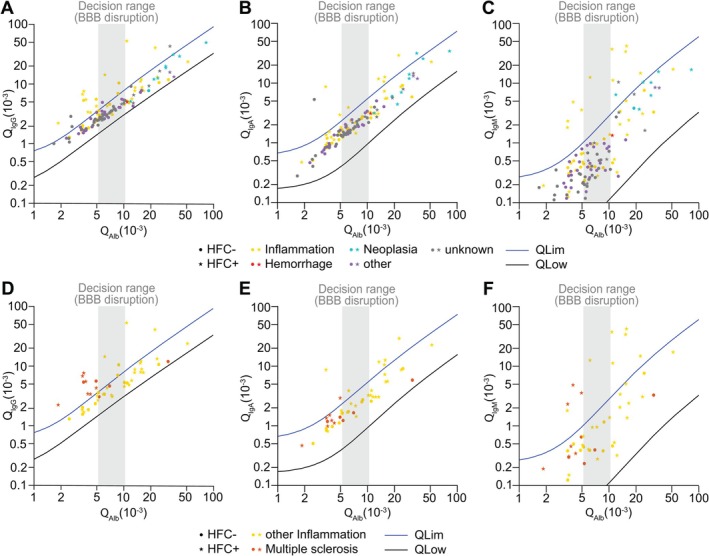
Diagnosis‐based analysis of CSF immunoglobulins comparing HFC+ and HFC– cases. Adapted Reiber diagrams for quotients of immunoglobulin (Ig) in CSF/serum (QIg) plotted against quotients of albumin levels in CSF/serum (QAlb) for all clinical categories (A–C) and for Inflammation only (D–F). (A, D) IgG, (B, E) IgA, (C, F) IgM. Dot represents HFC– cases, asterisk represents HFC+ cases; color of dots indicate clinical category with red, Hemorrhage; yellow, Inflammation; blue, Neoplasia; purple, other; grey, unknown; orange, multiple sclerosis. Blue line indicates upper levels of reference range for QIg (QLim); black line represents lower level of reference range for QIg (QLow). Grey area indicates age dependent decision range for blood–brain barrier (BBB) disruption.

Reiber diagrams, plotting the Ig quotient (Q_Ig_ of CSF/serum) against the albumin quotient (Q_Alb_ of CSF/serum), allow the differentiation of whether Ig entered the CNS by diffusion or was synthesized intrathecally. To test this issue in our HFC+ and HFC− groups and considering the five clinical categories, we adapted the well‐established Reiber diagram approach. For all three Ig plotted (IgG, IgA, IgM), there was a clear spatial separation within the Reiber diagram between the categories Neoplasia versus others and Neoplasia versus unknown (Figure 4A–C). Of those Neoplasia cases tested for intrathecal Ig synthesis (9/32; 28%), almost all were positive for HFC (89%) and exceeded the age specific reference range for BBB disturbance (100%), but most of them (90%) were within the reference range for specific Ig. The clinical categories of others and unknown were similarly placed within the Reiber diagram and thus Ig specific reference range, while the category of Inflammation was widespread across the Reiber diagram. However, a relevant number of cases with Inflammation exceeded the Ig‐specific reference range and were HFC+ cases. Interestingly, retrospective reevaluation of clinical information for those cases revealed that this group suffered from multiple sclerosis (Figure 4D–F).

### 3.7 Definition of Predictive CSF Parameters Using Machine‐Learning Methods

To investigate whether CSF laboratory parameters are suitable for the prediction of the five clinical categories described above, we tested multiparametric correlation for each clinical condition using a mathematical algorithm called support vector machine (SVM). Of note, other machine methods, including random forest, logistic regression, gradient boosting, and perceptron were considered and compared to SVM, demonstrating the best performance for SVM. The CSF parameters such as macroscopic appearance, TCC including automatically detected HFC (both cell in % and cell/μl), lactate, glucose, TP, as well as OCB findings were standardized and used as features for the SVM‐based classification. All collected data were considered for SVM, including demographics and symptoms.

Considering all five clinical categories, the SVM model identified indeed HFC (cell/μl) having, like bloody CSF appearance and TP, a strong positive correlation with Hemorrhage (Figure S3A). Further, HFC (cell/μl), like TCC and OCB type 2, had a positive correlation with Inflammation (Figure S3B) and HFC (cell in %) and lactate had a positive correlation with the Neoplasia group (Figure S3C). Note that HFC as cell in % and HFC as cell/μl account for slightly different cell populations (Figure S3).

### 3.8 Definition of an HFC‐Based Mathematical Model for Case Stratification

**FIGURE 5:**
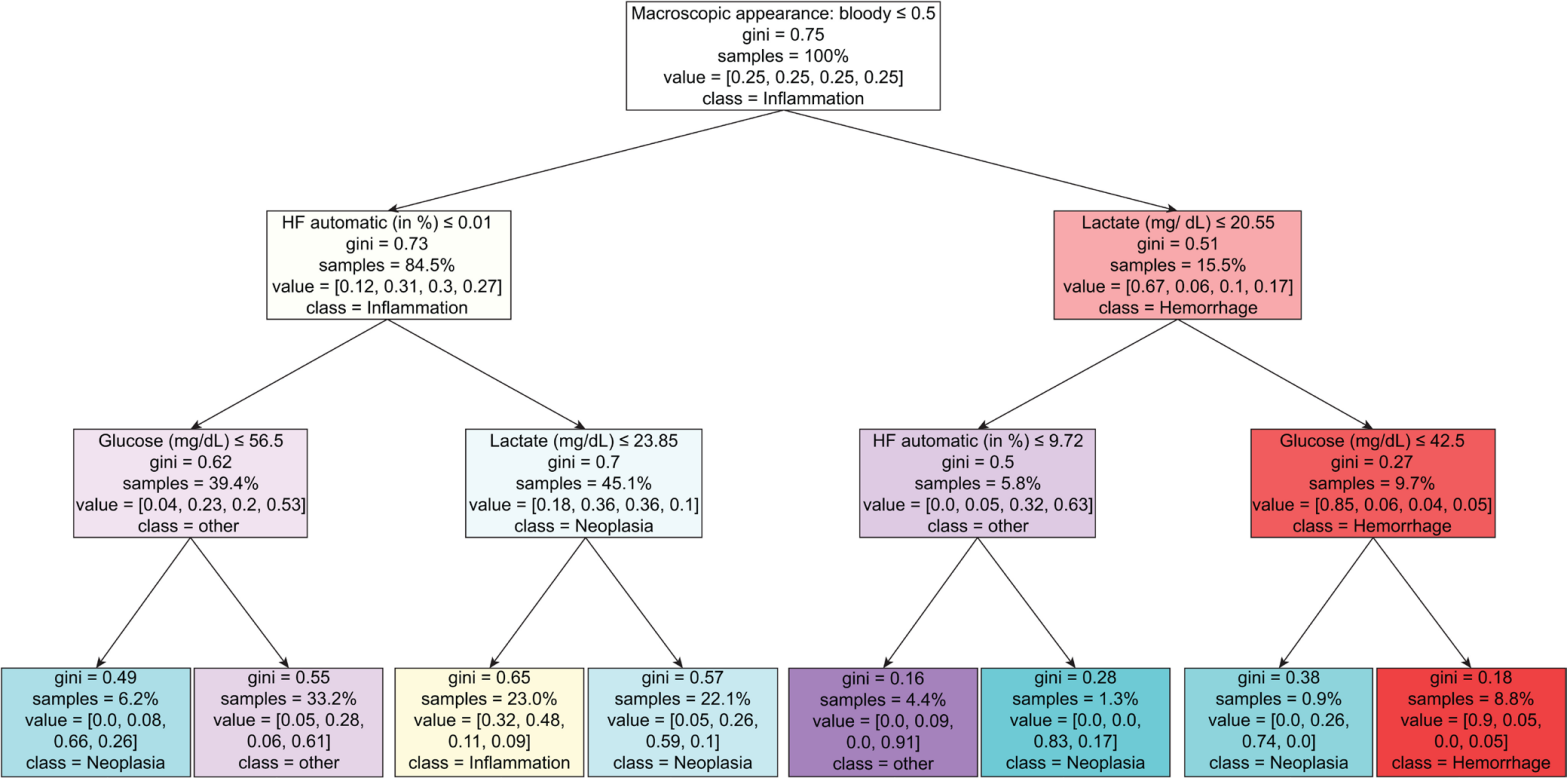
Multiparametric model for machine‐driven clinical case stratification. Graphic representation of the decision tree (*d* = 3). Color indicates clinical category: Red, Hemorrhage; yellow, Inflammation; blue, Neoplasia; purple, other. Color intensity correlates with the inverse of gini impurity. Gini is the probability of a randomly chosen element of a set being incorrectly labeled if it were labeled randomly and independently according to the distribution of labels in the set. The lower the gini impurity, the lower the likelihood of misclassification. Samples, the relative amount of samples included into analysis at each node in percent. Value, relative amount of clinical categories at each node [succession of clinical category: Hemorrhage, Inflammation, Neoplasia, other]. Class is the clinical category to which the cases would be classified at this node.

HFC from CSF may indeed be relevant for an appropriate stratification of cases into clinical categories: Hemorrhage, Inflammation, Neoplasia, and others. For testing this hypothesis, the mentioned CSF parameters (see above) were considered for developing a corresponding mathematical model called a decision tree. The resulting decision tree, with a depth of three, showed that automatically (Sysmex XN hematology analyzer) counted HFC (relative proportion, cell in %) were indeed selected (by the decision tree) as a crucial decision criterion for classifying cases into specific clinical categories (Figure 5). Mathematically mapping the cases into these four clinical categories was feasible with a low to moderate likelihood of misclassification, indicated by low Gini impurities (Gini score ≥ 0.3–≤ 0.5) and compared to a high Gini impurity of 0.75 at random classification. The decision tree was evaluated using the strategy of ten‐fold cross‐validation (CV) and AUROC scoring (one class vs. rest). In our study, this mathematical approach resulted in an overall performance accuracy of ~70% (CV score of 0.69), which indicates a moderate likelihood of correct case classification, compared to an expected random CV score of 0.25 for four different clinical categories.

In contrast to the decision tree including HFC into the data set (Figure 5), a decision tree without HFC, where HFC were discarded from the data set and thereby could not be selected as a decision point, selected the parameter cell count (TCC) for case stratification (Figure S4). This approach resulted in a CV score of 0.68. Note that a CV score of 0.25 is considered random.

## 4 Discussion

Automatic cytological examination of CSF by Sysmex XN‐Series hematology analyzers is a well‐established, convenient, and widely used laboratory approach not only for counting but also for differentiation of nucleated CSF cells into the three different subtypes MN, PMN, and HF. While MN and PMN cells already represent clinically well‐validated diagnostic laboratory parameters, HFC are still considered a research parameter, although some indications for associations in neurological and non‐neurological diseases exist [1, 11, 12, 13]. Therefore, we aimed to examine the diagnostic HFC relevance for specific neurological disease categories. We performed numerous correlation analyses considering established routine CSF (and serum) laboratory parameters and applied several methods of mathematical disease modelling to define the diagnostic usability of HFC.

Comparative cytological CSF analyses by manual microscopic cell differentiation, which still serves as a gold standard technique, and automatic cell detection by a hematology analyzer showed that a majority (~80%) of HFC represents plasma cells, macrophages, and/or malignant cells. However, in ~20% of cases automatically characterized as HFC+, we could not identify the HFC correlates (plasma cells, macrophages, malignant cells) and suspect that those cases feature meningothelial, ependymal, choroid plexus, and/or cartilage cells admixed to CSF due to the performance of lumbar puncture. In line, previous studies could demonstrate that automatic hematology analyzers sometimes classify both mesothelial and cartilage cells as HFC as well [14, 27, 29]. Even in manual microscopy, cartilage cells are sometimes misclassified as malignant cells [29]. Additionally, plasma cells might have been missed in the manual examination since the microscopic differentiation between borderline forms of activated lymphocytes and mature plasma cells is difficult [1] and has also turned out to be a major obstacle in automated image recognition applications [30].

The Bland–Altman analysis of automatic and manual CSF cell differentiation approaches for MN, PMN, and HF cells shows an appropriate method correlation for all five clinical categories and supports our hypothesis of diagnostic relevance of HFC. For a reliable interpretation of the Bland–Altman data, the range of two standard deviations should be evaluated for its clinical relevance [31]. However, the limits of agreement are sufficient to suggest that HFC values produced by the automatic method make it suitable for clinical use.

As most HFC were identified within the clinical categories Hemorrhage, Inflammation, and Neoplasia, we tested whether HFC positivity alone is sufficient to predict those clinical categories using the confusion matrix tool. Although most single cases from those groups demonstrated indeed the HFC, as shown by hit rates > 0.70, this positivity for HFC, on the other side is still inadequate for a proper case stratification, as reflected by a marginal prediction ranging from 0.13 for Hemorrhage to 0.16 for Neoplasia and 0.35 for the Inflammation group. Nevertheless, the absence of HFC in CSF samples is a reliable exclusion criterion, as indicated by high negative predictive values of 0.80 for Inflammation and > 0.90 for Hemorrhage and Neoplasia.

Larruzea et al. already addressed the problem of low clinical HFC specificity, but could circumvent it using a higher cut‐off value for HFC [32]. Another improvement strategy has been suggested by applying a multiparametric analysis for differentiation between bacterial meningitis and other causes of CSF pleocytosis [33]. We chose a comparable but extended strategy, performing comprehensive multiparametric correlation analyses followed by various mathematical disease modelling approaches. Our multiparametric analysis indicate that macroscopic CSF appearance, higher TCC, TP, and lactate CSF concentrations correlate with the presence of HFC in CSF samples. However, the presence of HFC did not show a significant correlation for most laboratory parameters when particular clinical categories were considered. This could be due to the low CSF sample number in most of our clinical groups. As TP levels were significantly higher in HFC+ cases, we decided to perform modified Reiber scheme‐based correlation analyses. Surprisingly, the group of Neoplasia cases was clearly separated from the categories other and unknown within the Reiber diagram. The category of Inflammation was widely distributed across the Reiber diagram; however, the subgroup of cases suffering from multiple sclerosis showed the tendency for higher IgG CSF levels while having low albumin CSF levels (sensitivity = 75%, specificity among inflammations = 100%). Interestingly, some of these cases were also positive for HFC, proposing that HFC in these cases most likely represent plasma cells.

Applying a mathematical method based on the SVM model, we were able to identify the relative proportion of HFC among a few other parameters, showing a strong positive correlation with at least four of five clinical categories. This was not the case when performing the multiparametric box plot‐based analysis. However, similar to the results from the boxplot‐based analysis for clinical decisions, the SVM‐based findings for HFC should be interpreted in a multiparametric context. Surprisingly, almost the same CSF parameter spectrum (bloody CSF appearance, HFC, glucose and lactate) has been identified by the mathematical model of the decision tree for predicting the clinical categories, and thus for case stratification. The likelihood of misinterpretation differed among the clinical groups, which was to be expected, as presumably the diagnostic significance of HFC is much higher in cases with suspected neoplasia than in cases with for example, unknown neurological disease. A benefit of the decision tree model is the definition of cut‐offs for relevant CSF parameters that could also be used in further clinical diagnostic settings.

There are several limitations to our study. First, the number of cases included is not particularly high. Second, our data were collected within a single‐center study and have not been replicated. Furthermore, an initial classification of the cases was made into five superordinate clinical categories, each covering a wide range of pathologies. Therefore, in extending analyses, it might be useful to define further specified clinically relevant groups for receiving better diagnostic evidence for our predictive model.

In sum, our findings show that HFC (together with other laboratory CSF parameters) has the potential for appropriate case stratification. Our decision tree algorithm with HFC, where HFC was included in the data set and selected as a decision point by the algorithm, classified almost 70% of cases correctly (CV score: 0.69) compared to an expected random CV score of 0.25 or a CV score of 0.68 when excluding HFC from the data set. The fact that total cell count was chosen as a replacement just proves the importance of HFC in a different way. In the HFC+ cases, total cell count was increased about 70 times. Accordingly, the majority of the total cell count corresponds to HFC/μl.

In a study reporting on a decision tree‐based classification to support Alzheimer disease diagnosis using CSF biomarkers, a CV score of ~0.75 was reported [34]. Note that comparing CV scores of decision tree models between studies is only possible to a limited extend. The model purpose, as well as cohort size, number of groups, data sets, etc., vary greatly from study to study, which will all impact CV scores. However, in this study, only three groups (random classification of 0.33) were present, which can be a reason for the higher CV values. Other comparable studies with two groups (random classification = 0.5) resulted in higher accuracy scores of 0.80–0.86 [35, 36].

Although the CV of 0.69 in our study seems not to be adequate for clinical practice yet, our approach holds promise for extended analyses in larger datasets. Therefore, our strategy described here could result in new supportive diagnostic tools for clinically relevant decisions.

Taken together, all these evidences suggest that HFC (positivity) could be routinely reported as a diagnostic laboratory parameter, but a further diagnostic step should lead to confirming diagnostics, like microscopic morphological differentiation. In perspective, HFC reporting can improve the effectiveness of patient diagnosis and treatment, resulting in cost savings and better patient care, especially when multiparametric laboratory diagnostic algorithms are used.

## Author Contributions

B.S., C.H., and A.J. analyzed and interpreted the data; M.P. contributed to data acquisition; B.S., K.F., and A.J. drafted the manuscript; B.S., C.H., and A.J. edited the manuscript; A.J., A.O., F.L.H., K.K., and K.R. contributed to the conception and design of the study. All authors critically revised the manuscript. All authors have accepted responsibility for the entire content of this manuscript and approved its submission.

## Ethics Statement

The study was approved by the ethics committee of the Charité—Universitätsmedizin Berlin (EA1/059/22) and the study was conducted in accordance with the Declaration of Helsinki (as revised in 2013).

## Consent

Informed consent was obtained from all individuals included in this study, or their legal guardians or wards.

## Conflicts of Interest

The authors declare no conflicts of interest.

## Supporting information


Data S1.

## Data Availability

The raw data can be obtained on request from the corresponding author.
