# Supplementary material for: Sysmex XN‐Based Evaluation of the Diagnostic Performance of High‐Fluorescent Cells From CSF as a Supportive Diagnostic Criterion in Neurological Diseases
Source: Int J Lab Hematol. 2025 Mar 20;47(4):632–42. doi: 10.1111/ijlh.14466 (PMC12239702; doi:10.1111/ijlh.14466)
Supplement: Supplementary file 1 — Data S1. [file IJLH-47-632-s001.docx]

**Supplementary Material**

**Table S1 Clinical categories**

| Clinical category | Diagnosis | HFC+ | HFC– |
| --- | --- | --- | --- |
| Hemorrhage | Hydrocephalus with hemorrhage | 1 | 0 |
|  | Intracerebral hemorrhage | 6 | 0 |
|  | Subarachnoid hemorrhage | 10 | 3 |
|  | Basal ganglia hemorrhage | 2 | 0 |
| Inflammation | Neurosurgical wound infection/ infected CSF cushion | 6 | 0 |
|  | Meningitis | 22 | 0 |
|  | Encephalitis | 3 | 2 |
|  | Meningoencephalitis | 2 | 0 |
|  | Myelitis | 1 | 0 |
|  | Spondylodiscitis | 2 | 1 |
|  | Inflammatory CSF syndrome | 1 | 0 |
|  | HIV encephalopathy | 1 | 0 |
|  | Neurosyphilis | 1 | 1 |
|  | Neuroborreliosis | 3 | 0 |
|  | Herpes zoster ophtalmicus/oticus | 9 | 0 |
|  | Neurotuberculosis | 0 | 1 |
|  | Neurosarcoidosis | 2 | 0 |
|  | Multiple sclerosis | 7 | 7^‡^ |
|  | Myasthenia gravis | 0 | 2 |
|  | Guillan-Barré syndrome | 0 | 1 |
|  | Temporal arteritis | 1 | 2 |
|  | Inflammatory disorders | 4 | 10 |
| Neoplasia | Meningeosis neoplastica | 6 | 0 |
|  | Primary brain tumor | 2 | 0 |
|  | Glioma | 1 | 0 |
|  | Meningioma | 2 | 0 |
|  | Lymphoma | 3 | 2 |
|  | Lung carcinoma | 2 | 1 |
|  | Sarcoma | 0 | 1 |
|  | Leukemia | 1 | 3 |
|  | Breast cancer | 2 | 0 |
|  | Renal cell carcinoma | 1 | 0 |
|  | Prostate cancer | 1 | 0 |
|  | Primary neuroectodermal tumor | 0 | 1 |
|  | Metastasis / other types of neoplasia | 3 | 0 |
| other | Pulmonary artery embolism | 0 | 1 |
|  | Sinus vein thrombosis | 1 | 0 |
|  | Cerebral ischemia / cerebral infarction | 5 | 10 |
|  | Intracranial hypertension | 1 | 3 |
|  | Epileptic seizure / status epilepticus | 1 | 14 |
|  | Chronic fatigue syndrome | 0 | 1 |
|  | Traumatic injuries | 1 | 1 |
|  | Headache (HaNDL syndrome, migraine) | 1 | 1 |
|  | Hydrocephalus | 5 | 3 |
|  | Peripheral polyneuropathy | 1 | 2 |
|  | Arachnoid cyst | 3 | 2 |
|  | Psychiatric disorders | 1 | 12 |
|  | Spinal muscular atrophies | 0 | 4 |
|  | Metabolic disorders | 2 | 0 |
|  | Orthopedic disorders | 0 | 4 |
| unknown | Unclear /no diagnosis | 9 | 28 |
|  | Headache of unknown origin | 1 | 2 |
|  | Reduced vigilance of unclear origin | 5 | 3 |
|  | Fever of unknown origin | 1 | 0 |
|  | Unclear mass | 2 | 0 |
|  | Sensory disorder of unclear origin | 0 | 5 |
|  | Facial palsy of unclear origin | 1 | 1 |
| Numbers represent the number of HFC+ and HFC– cases, respectively, for each diagnosis. ^‡^, two HFC– cases lacked information for Reiber schemes resulting in n=5 in the respective feature. | | | |

**Table S2 Origin of HFC samples**

| Medical field | Department | HFC+ | HFC- |
| --- | --- | --- | --- |
| Neurology/ Neurosurgery | Neurology | 50 | 87 |
|  | Neurosurgery | 43 | 10 |
|  | Interdisciplinary Neuro-Intensive care unit | 25 | 6 |
| Anesthesiology/ Surgical intensive care unit |  | 12 | 11 |
| Internal medicine | Hematology, Oncology, Tumor Immunology | 7 | 7 |
|  | Nephrology and Intensive medicine | 5 | 5 |
|  | Infectiology and Pneumology | 0 | 4 |
|  | Gastroenterology/ Infectiology/ Rheumatology | 2 | 1 |
|  | Rheumatology and Clinical Immunology | 0 | 1 |
|  | Endocrinology | 0 | 1 |
| Ophtalmology and Otorhinolaryngology |  | 2 | 2 |
| Radiation Oncology and Radiotherapy |  | 1 | 0 |
| Dermatology |  | 1 | 0 |
| Unclear | Emergency room | 1 | 0 |

**Table S3 Quantification of manual microscopic morphology analysis**

| Plasma cells | Plasma cells and macrophages | Macrophages | Macrophages and tumor cells | Tumor cells | Tumor cells and plasma cells | None of these |
| --- | --- | --- | --- | --- | --- | --- |
| 48 | 8 | 51 | 2 | 5 | 5 | 30 |
| Numbers represent the number of HFC+ cases having HFC classified into respective morphology group. | | | | | | |

**Table S4 Quantification of grouping of HFC+ and HFC– cases into clinical categories**

|  | Hemorrhage | Inflammation | Neoplasia | Other | unknown |
| --- | --- | --- | --- | --- | --- |
| HFC+ | 19 | 65 | 24 | 22 | 19 |
| HFC– | 3 | 27 | 8 | 58 | 39 |
|  | P=0.001 | P<0.001 | P=0.01 | P<0.001 | P=0.003 |
| Numbers represent the number of HFC+ cases grouped into respective diagnosis group. P-values of statistical testing for significance between HFC+ and HFC– using Chi square test. | | | | | |

**Table S5 Confusion matrix for diagnosis based on HFC positivity**

|  |  | Actual class | |  |
| --- | --- | --- | --- | --- |
| N=284 |  | Diagnosis positive | Diagnosis negative |  |
| Hemorrhage | | | | |
| Predicted class | HFC+ cases | TP: 19 | FP: 130 | PPV: 0.128 |
|  | HFC– cases | FN: 3 | TN: 132 | NPV: 0.978 |
|  |  | Sensitivity: 0.864  (95% CI: 0.651–0.971) | Specificity: 0.504  (95% CI: 0.442–0.566) | Accuracy: 0.532  (95% CI: 0.472–0.591) |
| Inflammation |  |  |  |  |
| Predicted class | HFC+ cases | TP: 65 | FP: 84 | PPV: 0.346 |
|  | HFC– cases | FN: 27 | TN: 108 | NPV: 0.800 |
|  |  | Sensitivity: 0.707  (95% CI: 0.602–0.797) | Specificity: 0.563  (95% CI: 0.489–0.634) | Accuracy: 0.609  (95% CI: 0.550–0.666) |
| Neoplasia | | | | |
| Predicted class | HFC+ cases | TP: 24 | FP: 125 | PPV: 0.161 |
|  | HFC– cases | FN: 8 | TN: 127 | NPV: 0.941 |
|  |  | Sensitivity: 0.750  (95% CI: 0.566–0.885) | Specificity: 0.504  (95% CI: 0.441–0.567) | Accuracy: 0.532  (95% CI: 0.4718–0.591) |
| N represent the number of total cases used for confusion matrices of each clinical category. TP, true positive; FP, false positive, FN, false negative, TN, true negative; PPV, positive predictive value; NPV, negative predictive value. 95% confidence intervals (CI) were calculated using the Clopper-Pearson exact method. | | | | |

**Table S6 Quantification of multiparametric assay**

| Feature (numerical) |  | HFC+^‡^ | n | HFC– | n | p-value | Difference (%) |
| --- | --- | --- | --- | --- | --- | --- | --- |
| Total cohort  (# patients) | Clear | 78 | | 122 | | <0.01* | -36.1 |
|  | Bloody | 34 | | 8 | | <0.01* | 325.0 |
|  | Turbid | 9 | | 2 | | 0.05* | 350.0 |
|  | Xanthochromic | 27 | | 3 | | <0.01* | 800.0 |
| Hemorrhage  (# patients) | Clear | 2 | | 1 | |  | 100.0 |
|  | Bloody | 12 | | 2 | |  | 500.0 |
|  | Turbid | 1 | | 0 | |  |  |
|  | Xanthochromic | 4 | | 0 | |  |  |
| Inflammation  (# patients) | Clear | 45 | | 27 | |  | 66.7 |
|  | Bloody | 5 | | 0 | |  |  |
|  | Turbid | 4 | | 0 | |  |  |
|  | Xanthochromic | 10 | | 0 | |  |  |
| Neoplasia  (# patients) | Clear | 14 | | 8 | |  | 75.0 |
|  | Bloody | 3 | | 0 | |  |  |
|  | Turbid | 2 | | 0 | |  |  |
|  | Xanthochromic | 5 | | 0 | |  |  |
| other (# patients) | Clear | 5 | | 48 | |  | -89.6 |
|  | Bloody | 8 | | 5 | |  | 60.0 |
|  | Turbid | 1 | | 2 | |  | -50.0 |
|  | Xanthochromic | 8 | | 3 | |  | 166.7 |
| unknown  (# patients) | Clear | 12 | | 38 | |  | -68.4 |
|  | Bloody | 6 | | 1 | |  | 500.0 |
|  | Turbid | 1 | | 0 | |  |  |
|  | Xanthochromic | 0 | | 0 | |  |  |
| Cell count/µL | total cohort | 354.6 ± 910.4 | 149 | 4.9 ± 8.9 | 135 | <0.01** | 7136.7 |
|  | Hemorrhage | 511.1 ± 924.0 | 19 | 20.0 ± 17.5 | 3 | 0.03** | 2455.5 |
|  | Inflammation | 407.2 ± 1127.7 | 65 | 6.1 ± 8.6 | 27 | <0.01** | 6575.4 |
|  | Neoplasia | 246.9 ± 615.2 | 24 | 4.5 ± 6.9 | 8 | <0.01** | 5386.7 |
|  | other | 340.6 ± 735.2 | 22 | 5.2 ± 10.7 | 58 | <0.01** | 6450.0 |
|  | unknown | 170.4 ± 487.0 | 19 | 2.5 ± 2.6 | 39 | <0.01** | 6716.0 |
| Glucose (mg/dL) | total cohort | 67.5 ± 33.4 | 149 | 74.4 ± 29.6 | 135 | <0.01** | -9.3 |
|  | Hemorrhage | 74.9 ± 14.9 | 19 | 68.0 ± 12.3 | 3 | 0.4** | 10.1 |
|  | Inflammation | 67.3 ± 42.9 | 65 | 87.9 ± 53.0 | 27 | 0.02** | -23.4 |
|  | Neoplasia | 59.1 ± 30.2 | 24 | 55.9 ± 9.2 | 8 | 0.7** | 5.7 |
|  | other | 65.9 ± 24.9 | 22 | 72.7 ± 16.6 | 58 | 0.1** | -9.4 |
|  | unknown | 73.4 ± 17.4 | 19 | 72.0 ± 22.9 | 39 | 0.5** | 1.9 |
| Lactate (mg/dL) | total cohort | 31.1 ± 21.5 | 149 | 17.1 ± 5.4 | 135 | <0.01** | 81.9 |
|  | Hemorrhage | 33.09 ± 14.4 | 19 | 22.7 ± 1.4 | 3 | 0.2** | 45.8 |
|  | Inflammation | 31.6 ± 26.0 | 65 | 17.1 ± 4.2 | 27 | <0.01** | 84.8 |
|  | Neoplasia | 38.2 ± 24.0 | 24 | 18.1 ± 6.7 | 8 | <0.01** | 111.0 |
|  | other | 25.7 ± 12.1 | 22 | 17.4 ± 6.1 | 58 | <0.01** | 47.7 |
|  | unknown | 23.6 ± 10.6 | 19 | 16.0 ± 5.0 | 39 | <0.01** | 47.5 |
| Total protein (mg/L) | total cohort | 1724.1 ± 2758.1 | 149 | 467.1 ± 314.8 | 135 | <0.01** | 269.1 |
|  | Hemorrhage | 3485.6 ± 4138.4 | 19 | 1124.1 ± 330.0 | 3 | 0.8** | 210.1 |
|  | Inflammation | 1380.4 ± 2027.1 | 65 | 576.6 ± 503.2 | 27 | <0.01** | 139.4 |
|  | Neoplasia | 2236.0 ± 3896.2 | 24 | 435.3 ± 214.4 | 8 | 0.01** | 413.7 |
|  | other | 1030.3 ± 1067.1 | 22 | 424.2 ± 244.6 | 58 | <0.01** | 142.9 |
|  | unknown | 1295.1 ± 2291.0 | 19 | 411.0 ± 153.2 | 39 | <0.01** | 215.1 |
| QAlb | total cohort | 16.1 ± 14.8 | 51 | 6.8 ± 4.8 | 76 | <0.01** | 136.8 |
| QIgG | total cohort | 11.5 ± 12.3 | 51 | 3.6 ± 2.3 | 76 | <0.01** | 219.4 |
| QIgA | total cohort | 7.3 ± 8.2 | 51 | 1.9 ± 1.5 | 74^‡‡^ | <0.01** | 284.2 |
| QIgM | total cohort | 16.5 ± 54.3 | 51 | 0.6 ± 1.0 | 74^‡‡^ | <0.01** | 2650.0 |
| Depicted values represent mean ± SD, if not indicated differently, and sample size (n). ^‡^, one HFC+ case (Inflammation category) lacked information for the macroscopic appearance resulting in n=148 (total cohort) and n=64 (Inflammation) in the respective feature. ^‡‡^, two HFC– cases lacked information for IgA and IgM resulting in n=74 for the respective feature. P-values represent: *, Barnard’s Exact Test; **, Mann-Whitney U rank test. # = number. | | | | | | | |

**Table S7 Statistical analysis of the HFC+ group**

| Feature | p-value |
| --- | --- |
| Cell count/µL | 0.8 |
| Glucose (mg/dL) | 0.6 |
| Total protein (µg/L) | 0.02 |
| Lactate (mg/dL) | 0.2 |
| HF automatic (in %) | <0.0001 |
| Depicted p-values represent ANOVA tests of HFC+ cohort and correspond to data presented in Figure 2 and 4. | |

**Supplementary Figure 1**

**
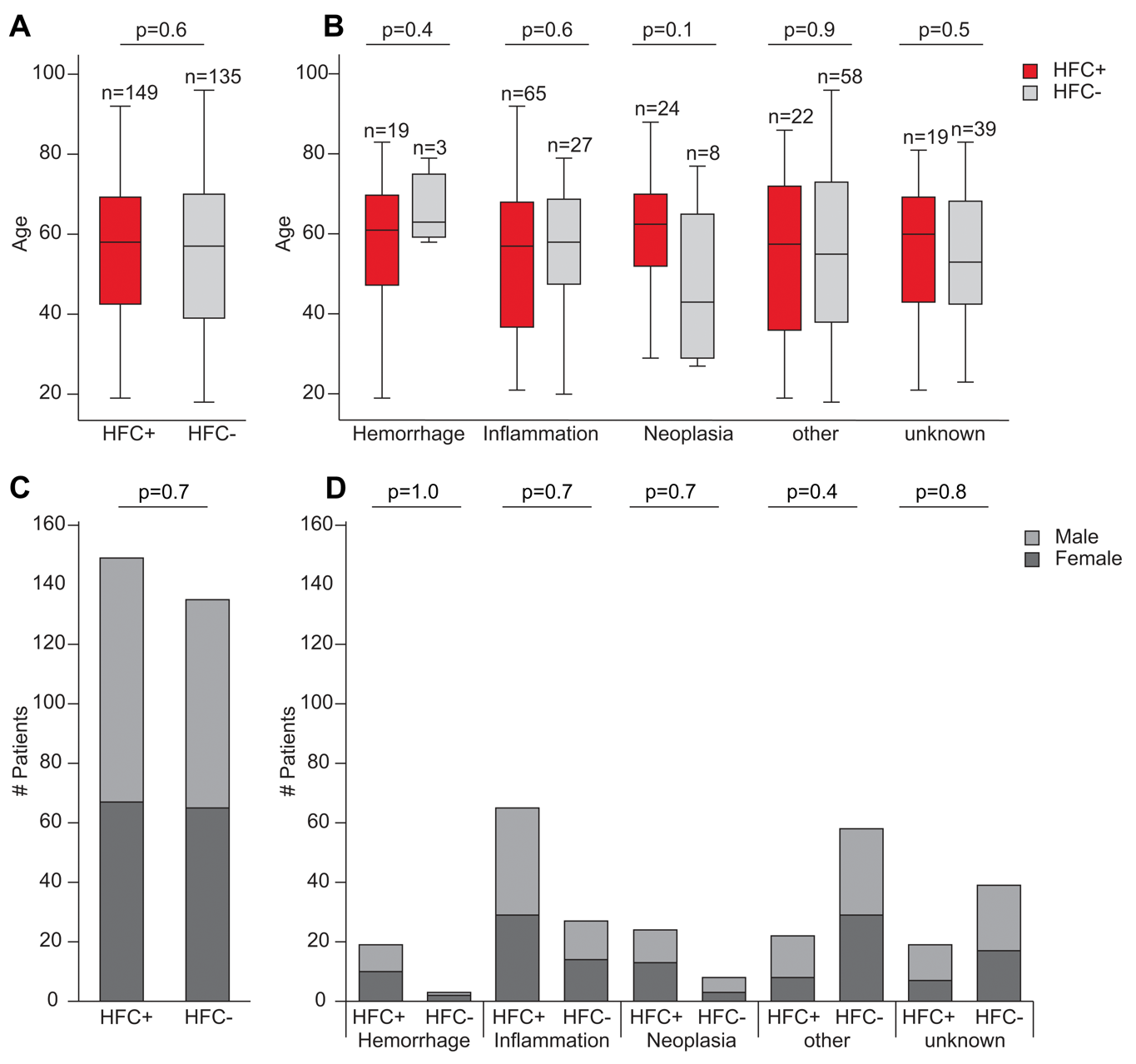
**

**Figure S1 Patient characteristics**

(A, B) Age differences of total HF cell positive cases (HFC+) compared to HF cell negative cases
(HFC–) (A) as well as of HFC+ compared to HFC– cases grouped into clinical categories (B). n represents number of cases per group, p represents statistical significance testing using Mann-Whitney U test.

(C, D) Gender differences of total HFC+ compared to HFC– cases (C) as well as of HFC+ compared to HFC– cases grouped into clinical categories (D). p, p-value of statistical significance testing using chi square test. # = number.

**Supplementary Figure 2**

**
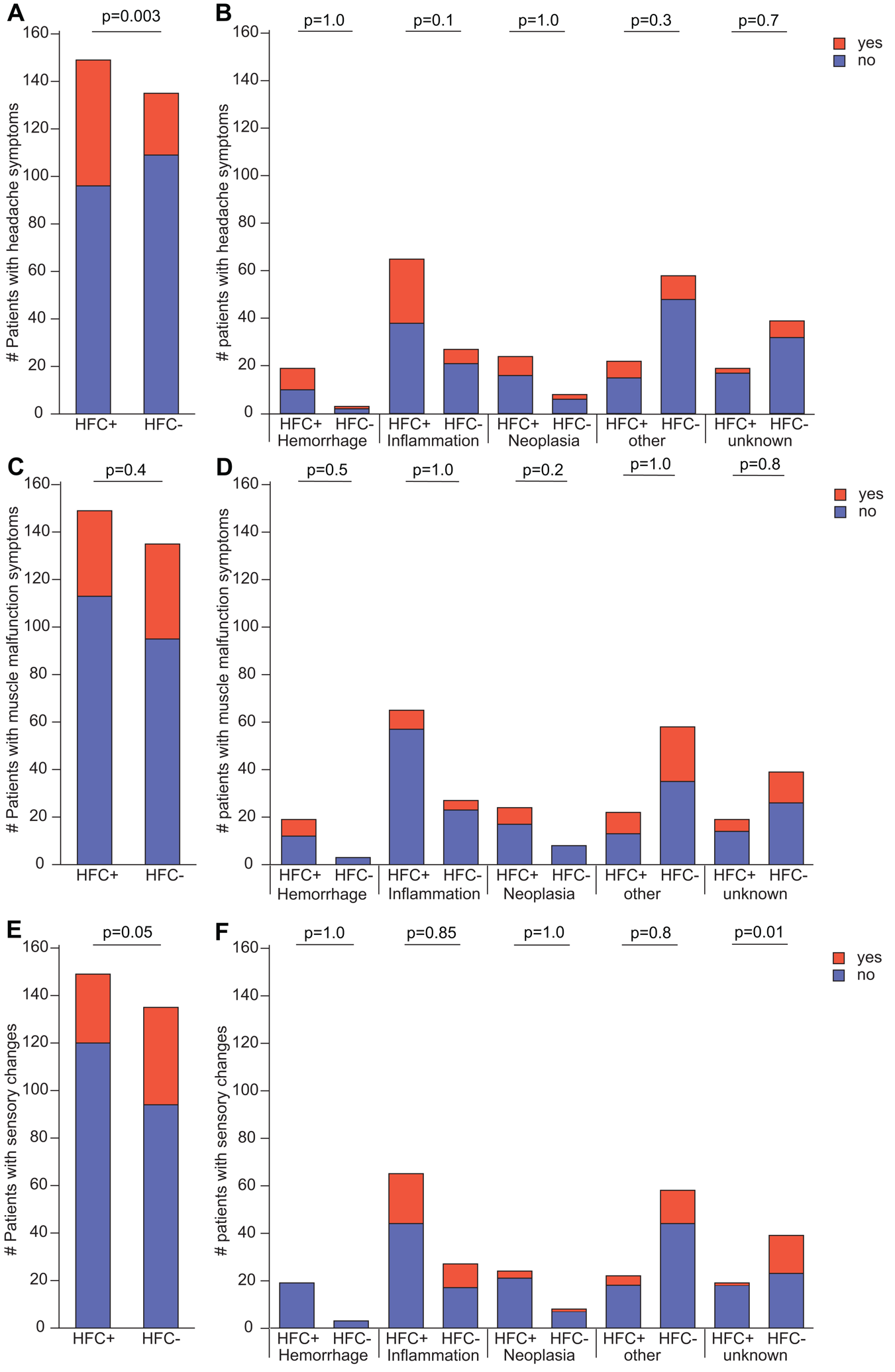
**

**Supplementary Figure 2: Changes in the presentation of symptoms in HFC+ compared to HFC– cases**

(A, B) Number of patients with headache symptoms in HFC+ and HFC– cases in total (A) as well as of HFC+ compared to HFC– cases grouped into clinical categories (B).

(C, D) Number of patients with muscle malfunction symptoms in HFC+ and HFC– cases in total (C) as well as of HFC+ compared to HFC– cases grouped into clinical categories (D).

(E, F) Number of patients with sensory changes in HFC+ and HFC– cases in total (E) as well as of HFC+ compared to HFC– cases grouped into clinical categories (F).

P, p-value of statistical significance testing using chi square test. # = number.

**Supplementary Figure 3**

**
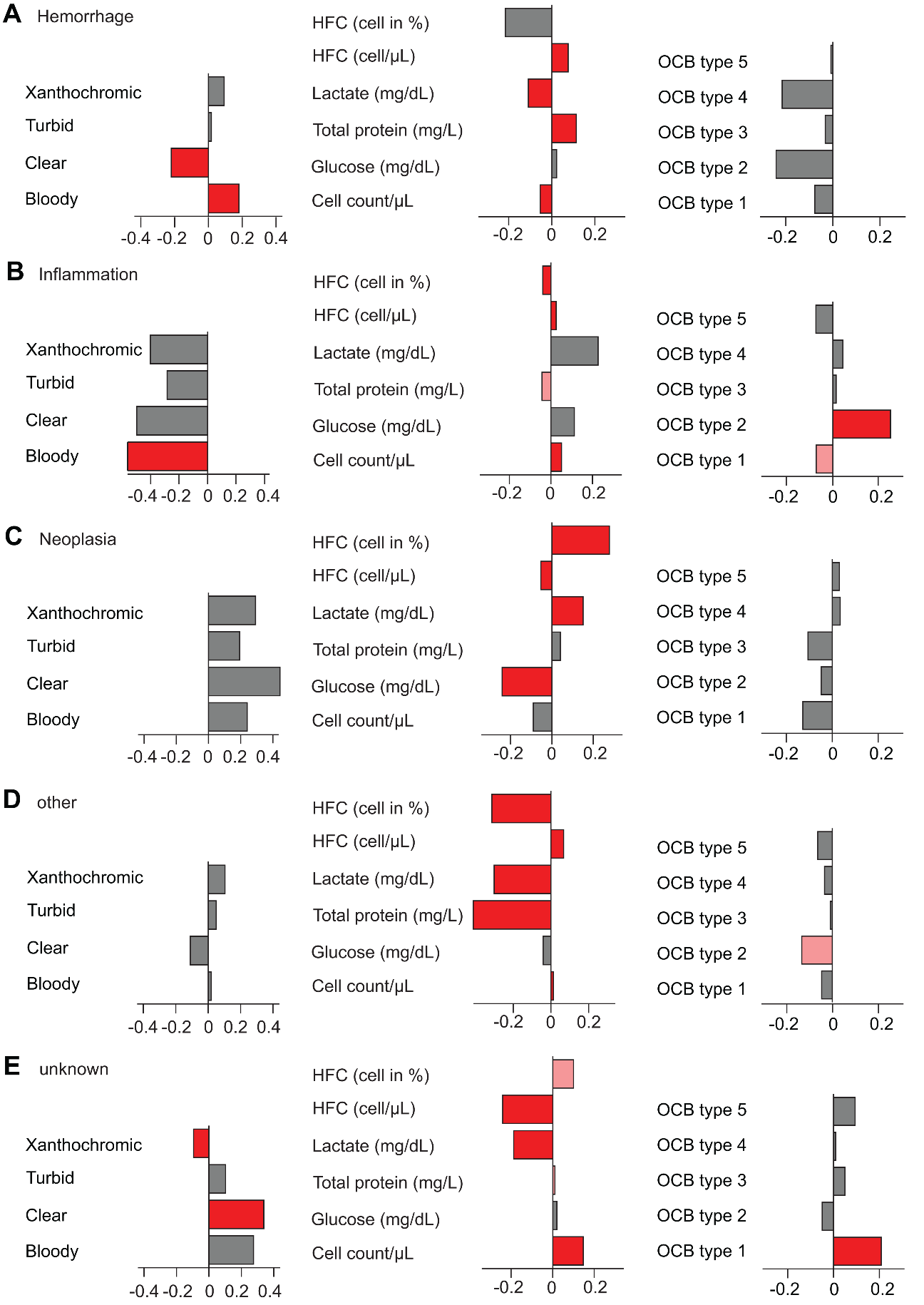
**

**Supplementary Figure 3** **Definition of potential predictive parameters by multiparametric analysis using support vector machine (SVM) model**

Schematic representations of the direction (positive/ negative) as well as significance of the correlation of the respective laboratory parameter with the respective clinical category based on the results of the multiparametric analysis for (A) Hemorrhage, (B) Inflammation, (C) Neoplasia, (D) others and (E) unknown. HFC as cell/µl represents the total HFC fraction including non-PMN and non-MN cell types, such as e.g. ependymal and/or mesenchymal cells. HFC as cell in %, in contrast to HFC as cell/µl, represents the relative fraction of HFC in the total population of only HFC, PMN, and MN. Grey, p≥0.05; light red, p≤0.05; red, p≤0.01 from Mann-Whitney U or Chi square, respectively. x-axis denotes respective feature importance in the SVM classifier, with values >0 supporting the model’s decision for the respective clinical category and values <0 against it. OCB, oligoclonal band.

**Supplementary Figure 4**

**
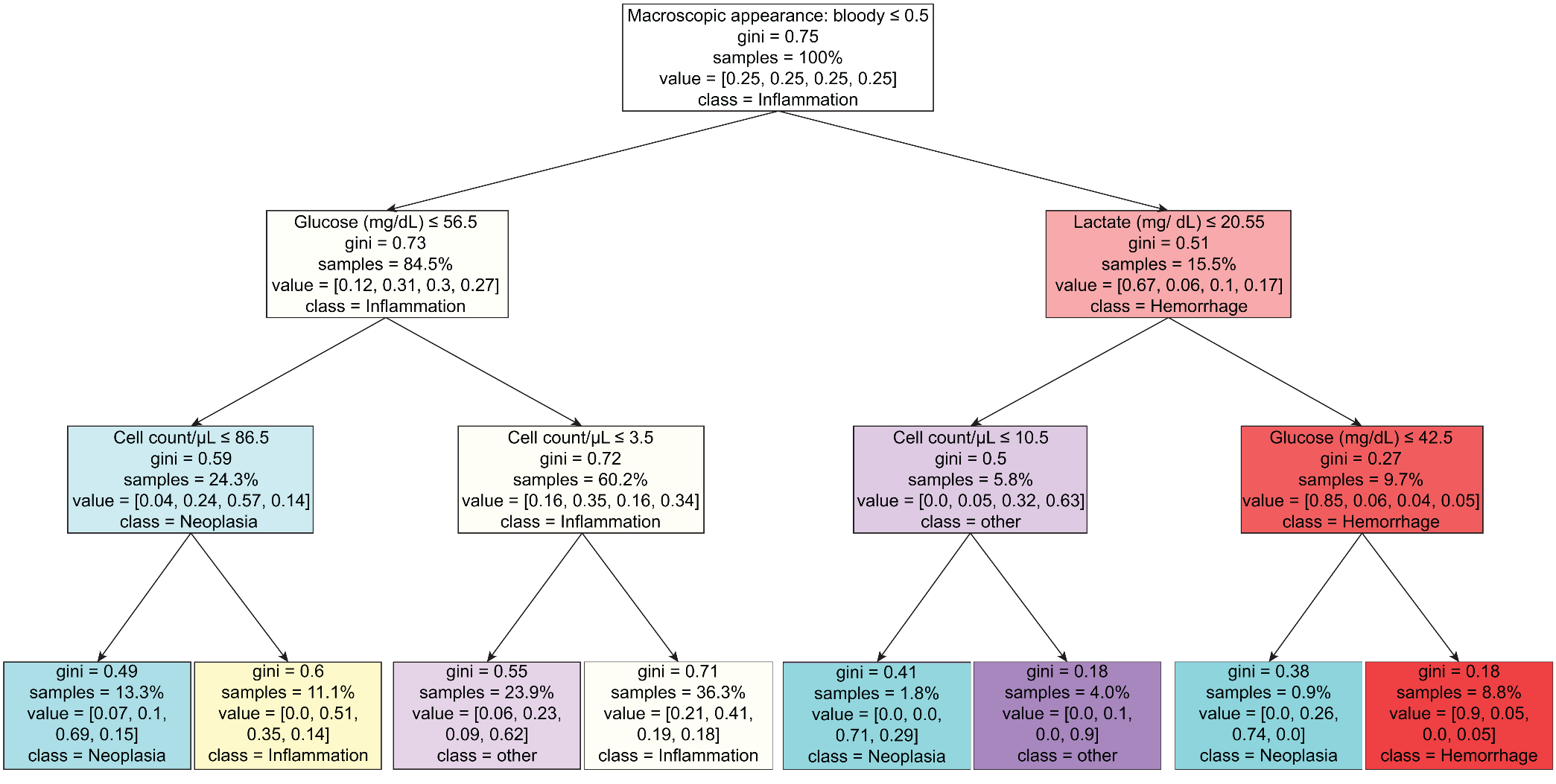
**

**Supplementary Figure 4 Decision tree without HFC**

Graphic representation of the decision tree without HFC in the dataset (d=3). Color indicates clinical category: red, Hemorrhage; yellow, Inflammation; blue, Neoplasia; purple, other. Color intensity correlates with the inverse of gini impurity. Gini, the probability of a randomly chosen element of a set being incorrectly labeled if it were labeled randomly and independently according to the distribution of labels in the set. Samples, the relative amount of samples included into analysis at each node in percent. Value, relative amount of clinical categories at each node [succession of clinical category: Hemorrhage, Inflammation, Neoplasia, other]. Class, clinical category which the samples/patients would be classified to at this node.
